# Supplementary material for: Emergence and control of photonic band structure in stacked OLED microcavities
Source: Nat Commun. 2021 Oct 20;12:6111. doi: 10.1038/s41467-021-26440-3 (PMC8528838; doi:10.1038/s41467-021-26440-3)
Supplement: Supplementary file 4 — Supplementary Data 1 [file 41467_2021_26440_MOESM4_ESM.zip › OLED Simulation v2-1/OLED Simulation/Materials Data/Materials Database/rii-database-2019-02-11.zip!/database/info/main/ZnSe.html]

# Zinc selenide, ZnSe

## External links

- Zinc Selenide - Wikipedia
- Zinc Selenide - WebElements
- Zinc Selenide - Infrared Multilayer Laboratory, University of Reading
- Zinc Selenide - II-VI Infrared
- Zinc Selenide - ICL
- Zinc Selenide - Crystran
